# A Framework to Assess Where and How Children Connect to Nature

**DOI:** 10.3389/fpsyg.2017.02283

**Published:** 2018-01-04

**Authors:** Matteo Giusti, Ulrika Svane, Christopher M. Raymond, Thomas H. Beery

**Affiliations:** ^1^Stockholm Resilience Centre, Stockholm University, Stockholm, Sweden; ^2^Department of Landscape Architecture, Planning and Management, Swedish University of Agricultural Sciences, Uppsala, Sweden; ^3^School of Education and Environment, Kristianstad University College, Kristianstad, Sweden

**Keywords:** assessment framework, child-nature-connectedness, human-nature connection, significant nature situations, nature routines, sustainable urban design, environmental education, mix-method approach

## Abstract

The design of the green infrastructure in urban areas largely ignores how people's relation to nature, or human-nature connection (HNC), can be nurtured. One practical reason for this is the lack of a framework to guide the assessment of where people, and more importantly children, experience significant nature situations and establish nature routines. This paper develops such a framework. We employed a mixed-method approach to understand what qualities of nature situations connect children to nature (RQ1), what constitutes children's HNC (RQ2), and how significant nature situations and children's HNC relate to each other over time (RQ3). We first interviewed professionals in the field of connecting children to nature (*N* = 26), performed inductive thematic analysis of these interviews, and then further examined the inductive findings by surveying specialists (*N* = 275). We identified 16 qualities of significant nature situations (e.g., “awe,” “engagement of senses,” “involvement of mentors”) and 10 abilities that constitute children's HNC (e.g., “feeling comfortable in natural spaces,” “feeling attached to natural spaces,” “taking care of nature”). We elaborated three principles to answer our research questions: (1) significant nature situations are various and with differing consequences for children's HNC; (2) children's HNC is a complex embodied ability; (3) children's HNC progresses over time through diverse nature routines. Together, these findings form the Assessment framework for Children's Human Nature Situations (ACHUNAS). ACHUNAS is a comprehensive framework that outlines what to quantify or qualify when assessing “child-nature connecting” environments. It guides the assessment of where and how children connect to nature, stimulating both the design of nature-connecting human habitats as well as pedagogical approaches to HNC.

## Introduction

Academic discourse is increasingly focused on how people see themselves in relation to nature (Ives et al., 2017). In the last few decades, academic interest in human-nature connection, or HNC, has spread from evolutionary explanations (Kellert and Wilson, 1993), to psychological constructs like ecological identity (Naess, 1973), nature relatedness (Nisbet et al., 2008) and inclusion with nature (Schultz, 2002), and across the scholarships of environmental and conservation psychology (Saunders and Myers, 2003; Gifford and Nilsson, 2014; Zylstra et al., 2014), landscape management (Lewicka, 2011; Meyfroidt, 2013), biological conservation (Miller, 2006; Simaika and Samways, 2010), and more recently urban design (Andersson et al., 2014; Marcus et al., 2016; Colding and Barthel, 2017). Despite the heterogeneity of approaches, this body of literature consistently reports two trends. First, a set of values and beliefs facilitates pro-environmental choices and behaviors (Black et al., 1985; Guagnano et al., 1995; Stern, 2000; Hunecke et al., 2001; Thogersen, 2005; Kaiser et al., 2011). Second, a deep-seated HNC is nurtured by direct nature interaction during childhood (Chawla, 1998, 1999; Kahn, 2002; Evans et al., 2007; Hsu, 2009) and is remarkably stable in adulthood (Kaiser et al., 2014). These two trends highlight the importance of early nature experiences in shaping psychological traits of HNC, and suggest that HNC has a role to play in the transgenerational establishment of sustainable futures (Matsuba et al., 2012; Conrad, 2017).

Approaches to sustainability science that analyze social-ecological systems have broadly discussed a shared societal mindset of connection to nature (Folke, 2006; Folke et al., 2011; Díaz et al., 2015; Fischer et al., 2015). In this line of research, social and ecological dynamics are conceptualized as components of a single integrated system in which structure, goals, and overall trajectory are shaped by society's mindset (Meadows, 2008). The encompassing concept of HNC has, therefore, been considered the strongest leverage point to transform or transition a social-ecological system toward a desirable, resilient, and sustainable future (Abson et al., 2017). It follows from all the considerations above that direct experiences of nature during childhood are fundamental moments of sustainable enculturation, with long-lasting consequences for sustainable social-ecological systems. Considering how the physical living environment of humankind, i.e., the human habitat, provides nature experiences for children is therefore a crucial step toward reaching sustainable developmental goals.

In 2014, 54 percent of humankind lived in cities, and by 2050, 66 percent of the world's population is projected to be urban (United Nations, 2014). Cities are, and increasingly will be, the most common human habitat. The urban green infrastructure, or its absence, will, therefore, be the leading background of children's direct experiences of nature. In this built landscape children will experience what nature is, what it is made of, how it works, and eventually create expectations for what nature should be in the future (Kahn, 2002). So, the obvious question to ask is: “is the urban nature designed to nurture children's HNC?” The short answer is no. Contrary to the standards advocated by the UN New Urban Agenda of “universal access to safe, inclusive and accessible, green and public spaces” (General Assembly, 2015, 22) the urbanization process largely ignores the design of nature experiences with measured penalties for public health (Lederbogen et al., 2011; Bratman et al., 2012; Hartig and Kahn, 2016), personal development (Chawla, 2015; de Keijzer et al., 2016), and the emotions, attitudes, and behaviors that define HNC (Giusti et al., 2014; Soga and Gaston, 2016). The absence of nature experiences in the human habitat is so severe that is also referred to as “extinction of experience” (Pyle, 1993) and it is an active concern in environmental education (Finch, 2008), environmental conservation (Miller, 2005; Samways, 2007; Simaika and Samways, 2010) and public health alike (Soga and Gaston, 2016). Overall, the urban space is far from being a human habitat that promotes a connection between its inhabitants and nature.

Two interlinked obstacles that hinder the design of “nature-connecting” human habitats come from an ontological separation between mind and body in modern urban design (Metzger, 2013). First, the dominant model of urban design is characterized by the top-down implementation of body functions, such as housing, working, recreation and transportation (Le Corbusier, 1943; Cities European Council of Town Planners, 1998). However, this linear and compartmentalized approach to urban living fails when challenged to address social and psychological requirements such as livability or HNC (Gehl, 2010; Portugali, 2011; Marcus et al., 2016). Modern urban design does not structure the relation between humans and environments beyond its material facets (Marcus et al., 2016; Samuelsson et al., 2017). Second, there are no tools to identify if the green infrastructure connects people to nature or not, nor to quantify or qualify where “extinction of experience” happens. There are indeed no standard criteria or guidelines to assess or categorize an environment as more or less “nature-connecting.” As a consequence, there is no possibility to even conceive what “connecting” environments could be or should look like in the design process. Questions like “where do people connect to nature?” and “what kind of ‘connecting’ nature experiences are missing from cities?” are fundamental to design green infrastructure that connects people nature, but they cannot be answered yet. The overarching purpose of this paper is to address this shortcoming.

In this paper we aim to develop a practical framework that defines criteria and guidelines that allow users to assess if an environment connects children to nature or not. We have been inspired by Heft's (1988) seminal work, in which he identified sets of suitable relations between children's behaviors and outdoor environments that have been consequently used to assess if an environment is more or less “child-friendly” (Kyttä, 2002, 2006). In support of this aim identify which sets of relations can categorize an environment as more or less “child-nature-connecting.” Classifying an environment as more or less child-friendly relies on quantifying or qualifying an environment using lists of behavioral and social criteria that are suitable for children. As of yet, a similar list of criteria to assess environments that nurture children's HNC does not exist. This paper fills this gap. Rephrasing the definition of the “obesogenicity” property of an environment (Swinburn et al., 1999), we define here “child-nature-connectedness” as “the sum of influences that the surroundings, opportunities, or conditions of life have on promoting human-nature connection in individuals or populations of children.” In other words, the properties that allow a social-ecological system to connect children to nature. The goal of this paper is, therefore, not to provide a tool that prescribes *how* to measure child-nature-connecting environments, but a framework that outlines the list of criteria of *what* ought to be measured. We note that in order to provide a useful framework any list of criteria should be sufficiently comprehensive of all relations that indicate children's HNC; transferable across different children and cultures; and of practical use for practitioners and researchers to identify where and how children connect with nature. Thus, we develop and test the framework for these properties to initiate the design of nature-connecting human habitats and inform educational programs that aspire to connect children to nature.

In the next section, we present the theoretical overview of HNC and the “child-nature-connecting” property of environment. We provide an overview of the concepts used and the three research questions in section Overview of Concepts and Research Questions. We then outline the methodology of this mixed-methods investigation, which involves an initial qualitative and inductive phase that informs a quantitative and testing phase. We then present the results and critically discuss them in relation to the goal of the paper.

## An embodied approach to human-nature connection and child-nature-connectedness

Despite an exponential growth in research on HNC in the last decade epistemological and ontological differences prevent the development of an instrument to assess child-nature-connectedness of environments using the existing literature. For example, HNC has been experimentally studied as an independent attribute of the mind, qualitatively observed and described through nature experiences elsewhere, and also investigated as a relationship between people and specific geographical locations (Ives et al., 2017). These co-existing bodies of literature are somewhat complementary, but unsuitable for comparison or integration with each other because of their fundamentally different epistemological traditions (Ives et al., 2017). For instance, “connectedness to nature” (Schultz, 2002; Mayer and Frantz, 2004), “nature relatedness” (Nisbet et al., 2008), and “environmental identity” (Clayton, 2003) are central conceptualizations of HNC in environmental psychology, but they can be difficult to unify with evolutionary conceptualizations of HNC (Kellert and Wilson, 1993; Beery et al., 2015) or theories of sustainability transformation (Manfredo et al., 2014; Abson et al., 2017). Additionally, the ontological nature of such literature presents further challenges to identify the important criteria of child-nature-connectedness.

Despite a variety of research approaches in HNC, the majority of studies operationalize a disembodied ontology of HNC, in which contextual factors are independent and often dismissed objects of investigation. An indication of the widespread use of such disembodied ontologies is that even though most studies specifically evaluate some form of personal HNC, the vast majority do not define what kind of nature people tend to connect to (Ives et al., 2017). Existing research has mostly investigated HNC as cognitive abstractions and attitudinal attributes, using an experimental approach that mostly ignores the role of people's body, culture, and environmental context (Gifford, 2014; Zylstra et al., 2014; Restall and Conrad, 2015; Lumber et al., 2017). Different disembodied conceptualizations of psychological HNC (Mayer and Frantz, 2004; Perrin and Benassi, 2009) have indeed been shown to be overlapping (Tam, 2013; Restall and Conrad, 2015) and with limited assessment capacity in real-world situations (Duffy and Verges, 2010; Ernst and Theimer, 2011; Bruni et al., 2015). Some have suggested that this limited capacity for real-world assessments derives from socio-physical de-contextualization (Duffy and Verges, 2010; Meyfroidt, 2013; Beery and Wolf-Watz, 2014; Restall and Conrad, 2015). A distinctive example of how de-contextualization affects real-world assessments is from two studies that used the same experimental design and methodology to assess “connectedness to nature” in two different locations and attributed opposite results to climate differences (Verges and Duffy, 2010; Bruni et al., 2012). The existing empirical evidence produced by disembodied conceptualizations of HNC and experimental approaches is often a deductive testing of theoretically-prompted concepts, and, therefore, has limited usefulness to construct a practical framework of assessment, which we aim to develop in this paper.

Limitations of disembodied operationalizations of HNC can be also found in environmental education. Since its inception with the Tbilisi declaration in 1977, a key goal of environmental education has been “to search for a new ethic based on respect for nature” (UNESCO, 1978, 28). However, the disembodied separation between body and mind assumed in environmental education has favored curricula that abound in ecological knowledge, but ignores practical skills and social circumstances (Hungerford et al., 1980). Despite environmental knowledge being widely available and promoted, children's experiences in nature has never been so rare (Soga and Gaston, 2016) and the ecological crisis never been so obvious. Environmental educators such as Nazir and Pedretti (2016) also recognized these limitations when they write:

“*For some time now, researchers and practitioners in the field of environmental education have been recommending a shift away from a focus on cognitive knowing about the environment toward raising peoples' environmental consciousness in deep and substantive ways (see, e.g., Gough, 1987; Gruenewald, 2004; Kahn, 2008; Bowers, 2009; Wals and Dillon, 2013). Bai and Romanycia (2013, 105) suggest that environmental consciousness raising is really about […] making ecological principles into habits of mind, body and heart […] creating spaces for multiple, meaningful interactions to take place (Wals and Dillon, 2013) and providing contextual embodied experiences (Greenwood, 2013)” (p. 288, 301)*.

To overcome the limitations shown above and investigate the criteria needed to assess child-nature-connectedness we then reject an ontological separation between mind and body and operationalize a relational, or transactional (Altman and Rogoff, 1987), approach based on affordance theory (Gibson, 1979) called embodied ecosystem (Raymond et al., 2017). The theory of affordances is a relational approach to human perception and behavior posited by Gibson (1979) that is defined by the relations that exist between humans' abilities and the features of the environment (Chemero, 2009). Traditionally, affordances have been assessed from a functional standpoint while ignoring the emotional dimensions that act as the motivational basis for action (Kyttä, 2003). A renowned example of this is Heft's (1988) list of children-outdoor relations mentioned above that inspired this research. However, “*Gibson hardly wanted to divide the world up into material, social, or cultural worlds, as he was against all division of environmental experience”* (Kyttä, 2002, 76) and in recent times we have seen attempts to include emotional (Roe and Aspinall, 2011) and social affordances (Kyttä, 2006) in assessment models of human-environment relations (Kyttä, 2006; Broberg et al., 2013). One of the latest formulations of affordance-based theory is the concept of embodied ecosystem, which highlights the relational values of ecosystems that dynamically emerge by the sets of relations existing between mind, body, culture, and environment (Raymond et al., 2017). By adopting the concept of embodied ecosystem in this research we move away from the identification of single measures of HNC abstractions. This ontological choice allows us to fully embrace the complexity of HNC, grasp the diversity of “connecting” nature experiences that might influence it, and appreciate how the relations between children's HNC and nature experiences jointly unfold across temporal, environmental, and cultural contexts.

## Overview of concepts and research questions

We use the term *significant nature situations* (SNS) here, instead of “connecting” nature experiences for two reasons. The first reason is to be coherent with existing literature. *Significant* nature experiences have long been used in the literature to denote those life experiences that “connect” people to nature (Tanner, 1980; Chawla, 1998, 1999, 2006; Stern, 2000). Second, we use *nature situations*, instead of nature experiences, to be consistent with the embodied ontological approach described in section An Embodied Approach to Human-Nature Connection and Child-Nature-Connectedness.

In order to create a framework to assess child-nature-connecting environments, we first need a list of criteria to identify what a significant nature situation is, and distinguish it from a non-significant one. It is self-evident that not all nature situations are equally significant to promote children's HNC. For example, climbing a tree influences children's HNC differently than climbing a cactus. It is, however, unfeasible to use a list of all nature activities that can possibly promote or hamper children's HNC to distinguish a significant nature situation from a non-significant one. Such list would be an endless catalog of all possible interactions with nature, including indoor and virtual ones (Kahn et al., 2009). Instead, we need to identify what are the distinguishing *qualities* reoccurring across different nature situations that connect children to nature. In other words, we first need to identify the *qualities of significant nature situations*. If we assume for example that “enjoyment” is a hypothetical quality of SNS, and that enjoyment can be assessed while a child climbs a tree, then it means that the child is in a nature situation significant for developing her HNC. Also, it is likely that such a nature situation will change how she will perceive climbing trees in the future. Thus, a framework to inform the assessment of significant nature situations has to include qualities of SNS as much as their relation to children's HNC over time. There are, therefore, three crucial components required for an assessment framework: it should identify what the qualities of SNS are; what constitutes children's HNC; and provide an indication of their relation over time. Consequently, we ask the following research questions:

RQ1. What are the qualities of significant nature situations?
RQ2. What constitutes children's human-nature connection?
RQ3. How do qualities of significant nature situations and children's human-nature connection relate to each other over time?

## Methodology

Since the goal of the paper is to define a framework useful to assess child-nature-connectedness of environments we adopted a “sequential exploratory research design for instrument development” (Creswell and Clark, 2007). As in our case, this mixed-method methodology was required to develop and test an instrument of classification that is not yet available (Creswell and Clark, 2007). The empirical work is characterized by two sequential phases. In phase 1 (qualitative), we performed semi-structured interviews with a pool of professionals in the field of connecting children to nature to inductively unveil the qualities of SNS (RQ1), what constitutes children's HNC (RQ2), and how qualities of SNS and children's HNC relate to each other over time (RQ3). In phase 2 (quantitative), we tested the results of phase 1 with an online survey that we distributed among a broader pool of professionals in the field of connecting children to nature. Here, we examined the results for comprehensiveness, transferability, and practicality and further explored the relations between qualities of SNS and children's HNC.

### Why a focus on professionals' expertise?

Practitioners that aim to connect children to nature are an international and very heterogeneous group of specialists who have developed the ability to design, perform, and assess nature-based activities for children throughout their professional careers. We chose to focus on these professionals because their practices, outlooks, and educational strategies are directly based upon a holistic understanding of children's HNC. Our research is therefore based on professionals from a diverse number of organizations and from a wide array of countries. Tapping into their expertise ensured comprehensiveness of different approaches and intents, sufficient transferability across children's ages and groups, and practical usability for a wide audience of actors.

Additionally, focus on professional expertise aligned the methodology of this investigation with the relational ontology of embodied ecosystems. Understanding which qualities distinguish significant nature situations from non-significant ones requires the observation of children's HNC as it unfolds in their actions, or changes over long periods of time. Professionals who observe children daily are capable of recognizing these patterns of change. We therefore relied on decades of professional observations and insights, rather than attempting an inevitably partial and potentially theoretically biased observation ourselves.

### Phase 1: identification of HNC and qualities of SNS

We used semi-structured interviews to question practitioners (*N* = 26; male = 5; female = 21) in the field of connecting children to nature in two consecutive steps. The first pool of practitioners (*N* = 11) was chosen to represent a wide range of professional competencies and complementary conceptualizations of children's HNC. A second set of practitioners (*N* = 15) was selected from within a Swedish organization of nature preschools (“I Ur och Skur”) whose outdoor-focused pedagogical approach has contributed to increase interest and attachment to nature in young children through direct nature interaction since 1983 (Westerlund et al., 2016). With this second pool of interviewees (see Svane, 2017 for full report) we questioned, clarified, and deepened previously acquired information. All interviewed practitioners had between 5 and 40 (*M* = 18) years of expertise in the field so we refer to them in the paper as “professionals.” All interviewees provided informed consent prior to the interview. The interviews lasted 50 min on average and followed interview guidelines that covered the three main areas of interest identified by the RQs: what constitutes a “connecting” nature experience for children, what the traits of a connected child are, how children's HNC changes over time. The interviews were recorded, transcribed, and coded using Atlas.ti. Inductive coding was performed for all interviews following the systematic process for thematic analysis described by Braun and Clarke (2006).

The resulting themes denoting qualities of SNS (RQ1), children's HNC (RQ2), and their relationship over time (RQ3) were discussed between researchers and interpreted according to the objectives and the relational approach of the paper. We differentiated between themes when they were critically dissimilar and aggregated them when their differences were only of terminological nature. For example, when resulting themes were positive or negative manifestations of the same concept we aggregated them into an overarching theme. When the resulting themes were overlapping we highlighted their diversity whereas for nested themes we retained the most general. These inductive results were used to formulate the criteria of the assessment framework, which were then examined further by additional professionals using an online survey in phase 2.

### Phase 2: examining phase 1 results for comprehensiveness, transferability, and practicality

The online survey was developed following the process identified by Artino et al. (2014) to design high-quality survey scales. With the survey, we tested the criteria obtained from phase 1 and examined their comprehensiveness, transferability, and practicality. The survey was composed of four pages. The first page provided information about the study and survey, and ensured that all respondents gave informed consent for us to anonymously utilize the results for research purposes. The second page asked professionals for some descriptive information (e.g., number of years of professional experience in the field, the age of the children they work with). The third page addressed relevance and comprehensiveness of the constituents of children's HNC obtained in phase 1. First, participants were asked if each constituent indicated some form of children's HNC using a five-point Likert scale from “strongly disagree” to “strongly agree.” Professionals were then asked how comprehensive the list of *all* possible constituents of children's HNC was, and subsequently asked to rank all the constituents from the one children learn first to the one children learn last (hereinafter called *ordering exercise*). The fourth page examined the practicality and potential transferability of these constituents across a multitude of actors. In this exercise, we asked respondents to choose one constituent of children's HNC, to state a nature activity they perform in their everyday work to significantly nurture that constituent, and then to assess the reported significant nature activity rating how important each quality of SNS obtained in phase 1 was on a five-point Likert scale ranging from “not important” to “essential” (hereinafter called *assessment exercise*). Professionals were then asked to perform the same assessment exercise for another constituent of children's HNC, and were allowed to further perform it a third time.

We chose the number of questions (14 were compulsory) in the survey to be practical and simultaneously adequately comprehensive to capture the complex essence of children's SNS and HNC. We favored simple English vocabulary to suit the intended international audience of professionals working with children's HNC. Prior to releasing the survey, we conducted expert validation with four colleagues for content validity; eight cognitive interviews with additional professionals in the field for response process validity; and performed two phases of pilot testing (Artino et al., 2014). These practices ensured that the professionals responding to the survey understood and interpreted the final version of the questions and items correctly. The survey was individually emailed to professionals in the field of connecting children to nature in several countries. In addition, we also asked participants for further contacts.

We used R software for the analysis of the data. Specifically, the package *stats* and *likert* for the statistical analysis, the *daisy* function with Gower's coefficient for the analyses of dissimilarity, and the *agnes* and *hclust* functions of the package *cluster* for hierarchical clustering and dendrogram construction.

## Results

### Descriptive analysis of survey respondents

The 275 respondents of the survey were from more than 200 different organizations. These organizations included primary, secondary and high schools, forest and gardening schools, national parks, local and national organizations of environmental conservation, municipalities, and international organizations devoted to the cause of connecting children to nature. These organizations and schools were located in Sweden, Finland, Netherlands, US, England, Australia, Canada, Colombia, New Zealand, Scotland, Wales, Spain, Norway, Northern Ireland, Belgium, Mexico, Malaysia, India, Indonesia, Austria, Estonia, and Portugal. Only the data from respondents that had stated that connecting children to nature is either “very important” (*N* = 57) or “essential” (*N* = 218) for their profession were analyzed. This resulted in the removal of only 7 respondents suggesting that the survey was correctly targeted. Of all respondents 93% had at least 3 years of professional experience in the field of connecting children to nature (*M* = 15.5, Mdn = 12, max = 50, *SD* = 11), so, as with the interviewees in phase 1, we considered the respondents of the survey “professionals” in the field. Additionally, their heterogeneous professional competencies and institutions covered the full-spectrum of potential insights into what SNS are. Most professionals worked with several age groups at the same time. Only few respondents (13%) worked with the age group “0 to 1 years old,” 37% worked with “2 to 3 years old,” most of them (80%) worked with the age group “4 to 7 years old,” while 53% worked with “8 to 11 years old,” and 34% worked with “12 to 18 years old.”

The results from the interviews and survey are presented below alongside each other according to the research question they jointly explored and tested.

### RQ1: what are the qualities of significant nature situations?

From the inductive analysis of interviews with professionals we obtained a list of qualities that characterizes a nature situation with the potential to connect children to nature; that is, we identified the qualities of SNS. Hence, a SNS for children is characterized by one or more of the qualities listed in Table 1.

**Table 1:** List of qualities of significant nature situations with associated brief descriptions.

**Qualities of SNS**	**Brief description**
Entertainment	Nature situations that are fun, joyful, amusing, or enjoyable.
Thought-provocation	Nature situations that create new ways of conceiving human-nature interaction.
Intimacy	Nature situations that are private or intimate and allow a personal experience with nature.
Awe	Nature situations that are amazing, of overwhelming attraction, or mesmerizing, that create a “wow effect.”
Mindfulness	Nature situations that grasp children's focus and alertness, that make children “be in the flow.”
Surprise	Nature situations that are unpredictable or unexpected. In these nature situations children's line of thought is interrupted, and nature draws their attention.
Creative expression	Nature situations that involve arts, myths, stories, music, or role-play.
Physical activity	Nature situations that require body movement or any form of physical activity.
Engagement of senses	Nature situations that activate children's senses (smell, touch, hearing, etc.)
Involvement of mentors	Nature situations that involve persons, such as teachers, experts or relatives, who are capable of inspiring, encouraging, or leading the nature experience for the child.
Involvement of animals	Nature situations that involve interaction with animals.
Social/cultural endorsement	Nature situations that involve positive peer pressure, support from significant others, social acceptance, or cultural reinforcement.
Structure/instructions	Nature situations characterized by a set of rules that define the frame within which the child can act.
Child-driven	Nature situations that are chosen by the child, child-initiated (children autonomously decide when to begin the nature activity), and open-ended (children autonomously decide when to conclude the nature activity).
Challenge	Nature situations in which children overcome psychologically or physically adverse conditions, such as fear or cold.
Self-restoration	Nature situations of psychological, physical, or social relief. For example, relief from stress, fatigue, or gender stereotypes.

The descriptions presented here were also available to the respondents of the survey. The table can be read as, “A significant nature situation is characterized by (quality of SNS).”

**Figure 1:**
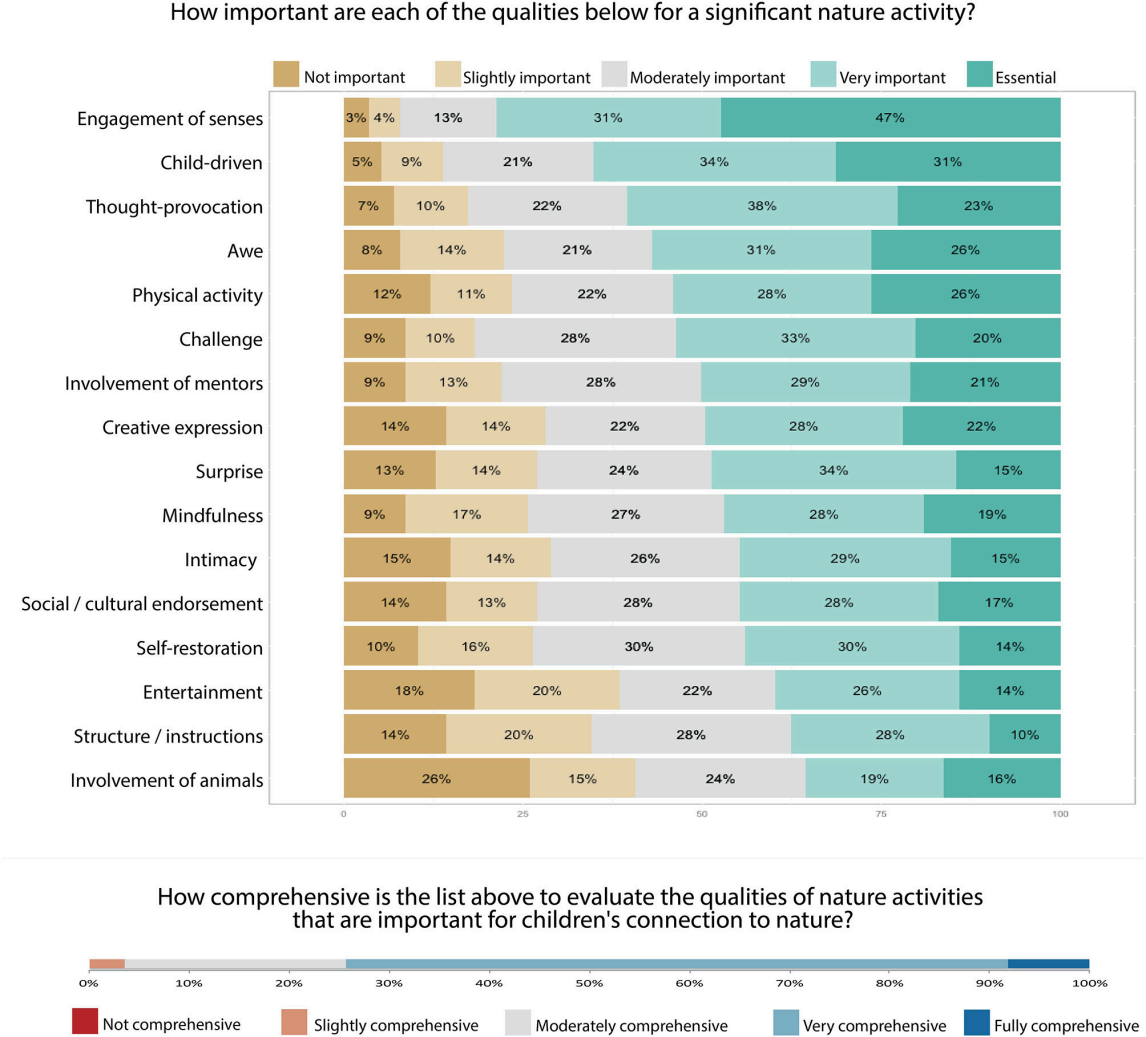
Results from the questions on qualities of SNS in the survey. **Top:** percentages of importance for all the qualities of SNS that professionals had used in assessing the significant nature activities they reported to perform with children. **Bottom:** professionals' responses on how comprehensive the list of qualities of SNS was for the assessment exercise.

During the assessment exercise in the survey, professionals reported 399 significant nature activities. These significant nature activities were often as simple as “pond dipping,” “lighting a fire,” “bug hunting” and “playing in the forest and parks,” but sometimes they were more complex; for example, “group sessions to talk about the permaculture ethics of earth care, people care and fair share” and “growing a vegetable garden using children's ideas.” All qualities of SNS were considered *very important* and *essential* to assess at least some of the 399 significant nature activities that the professionals reported (Figure 1). Of all respondents, 67% found the list of qualities of SNS *very comprehensive*, 7% *fully comprehensive* whereas no respondent found the list *not comprehensive*.

**Figure 2:**
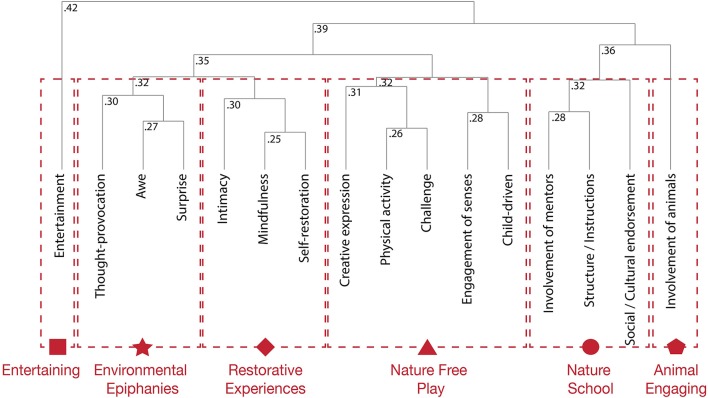
Dendogram obtained from the hierarchical analysis of dissimilarity of qualities of SNS using Gower's coefficient with height coefficients. The clusters of “entertainment” and “involvement of animals” had been independently clustered as “entertaining” and “animal engaging.” The remaining qualities of SNS were clustered into four different macro categories of nature situations: environmental epiphanies, restorative experiences, nature free play, and nature school.

In order to appreciate the differences and similarities in how qualities of SNS were used to assess significant nature activities we calculated a dissimilarity matrix using Gower's coefficients (min = 0.26, Mdn = 0.36, *M* = 0.36, max = 0.52) and subsequently performed hierarchical clustering. The dendrogram (agglomerative coefficient = 0.30) and clustering of the qualities of SNS at this level showed six separated clusters (Figure 2). Significant nature activities characterized by “thought-provocation,” “awe,” and “surprise,” were clustered together and named “*environmental epiphanies”*; nature activities characterized by “intimacy,” “mindfulness,” and “self-restoration” were clustered together and named “*restorative experiences”*; nature activities characterized by “creative expression,” “physical activity,” “challenge,” “engagement of senses,” and “child-driven” were clustered together and named “*nature free play”*; nature activities characterized by “involvement of mentors,” “structure/information,” and “social/cultural endorsement” were clustered together and named “*nature school*.” The hierarchical clustering showed that “entertainment” and “involvement of animals” were categorically different qualities that seemed to have intrinsic value and were independently clustered as “*entertaining*” and “*animal engaging*.”

### RQ2: what constitutes children's HNC?

The main themes emerged from the inductive analysis of the interviews showed that professionals recognized HNC as an ability that children manifest when they perform certain actions or show certain emotions. This was particularly evident when professionals compared children who had been taking part in nature activities for a long time with children that had just begun. We selected few illustrative quotes to exemplify this point.

“*We have one group that we have been working with for quite a long time, and […] then I have started with a new group, and then this thing is very clear […] they don't all have this feeling about how to think, or like: ‘before I start climbing this little mountain, I need to look where to go. I can't just go this way because it will be too steep, I need to go around’.”*

“*A couple of the… stark differences we notice in our children […] it takes them about 2 weeks on average to open up to the idea that they can also get dirty and wash up after, and it becomes something that's not quite so scary for them.”*

“*The ones who have been through this, they are quicker and faster and… they move differently. And that's the same when we go ice-skating or skiing, that the ones who have done this from when they were little, they have something in their body that makes it easier for them.”*

The main themes obtained from the inductive analysis of the interviews form a list of abilities that, according to the professionals, were clear indicators of children's HNC (Table 2). We called these indicators *abilities of human-nature connection*. Hence, a child more or less connected to nature is characterized by one or more of the abilities of HNC listed in Table 2.

**Table 2:** List of abilities of human-nature connection with the associated brief descriptions.

**Abilities of HNC**	**Brief description**
Feeling comfortable in natural spaces	The child demonstrates ease in natural spaces and feels comfortable with natural elements in the outdoors (e.g., dirt, mud, rain, or the sun).
Reading natural spaces	The child is able to see the possibilities for action in natural spaces that are not purposefully designed by man.
Acting in natural spaces	The child is able to perform activities in nature, for example, nature playing, camping, or outdoor sports in nature.
Feeling attached to natural spaces	The child shows a sense of belonging to specific natural spaces, to which they feel part of.
Knowing about nature	The child demonstrates knowledge of animals, plants, and ecological dynamics.
Being curious about nature	The child shows interest and motivation in exploring nature.
Recalling memories with nature	The child is able to recall past nature experiences and tell stories of lived experiences with nature.
Taking care of nature	The child is able to be responsible for nature and feels empowered to act for the wellbeing of nature.
Caring about nature	The child is able to feel care, concern, sensitivity, empathy, and respect for nature.
Being one with nature	The child is able to identify with nature and has a sense of profound personal attachment to nature that can be described as spiritual. Love for nature, humbleness in relation to nature, and assuming to be a small part of the immensity of nature are manifestations of this ability.

The descriptions presented here were also available to the respondents of the survey. The table can be read as, “A child connected to nature is capable of (abilities of HNC).”

**Figure 3:**
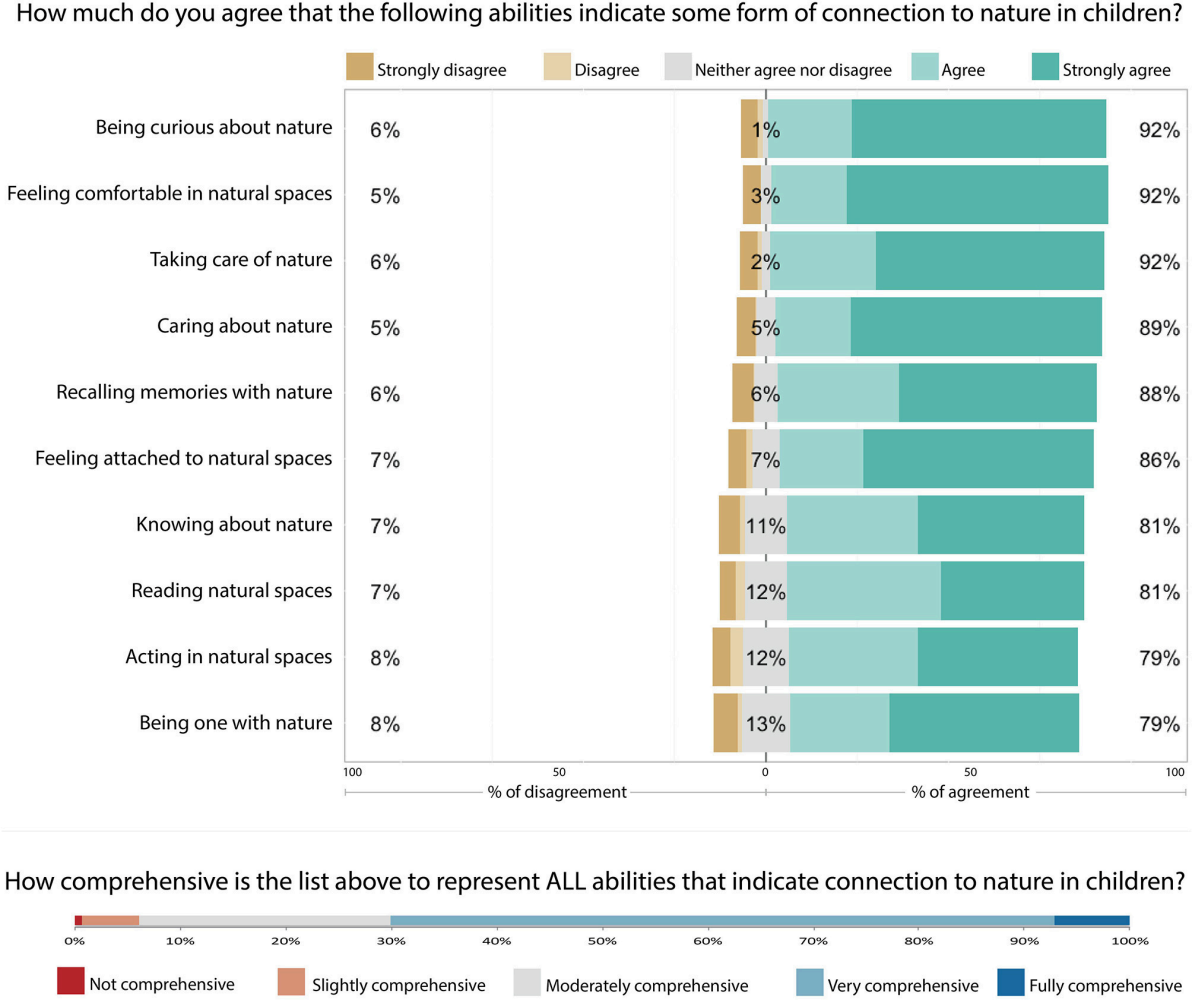
Results from the questions on abilities of HNC in the survey. **Top:** how much professionals agreed that the list of abilities of human-nature connection indicated children's HNC. **Bottom:** professionals' responses on how comprehensive the list of was to represent all abilities that indicate children's HNC.

Across all abilities of HNC, at least 79% of survey respondents *agreed* or *strongly agreed* that the abilities shown indicated some form of HNC in children, whereas *disagreement* ranged from 5 to 8% (Figure 3). Furthermore, only 0.7% of the respondents stated that the list of abilities of HNC was *not comprehensive*, whereas all others stated that the list of abilities of HNC was either *slightly* (5%), *moderately (24%), very* (63%), or *fully comprehensive* (7%).

### RQ3: how do qualities of significant nature situations and children's human-nature connection relate to each other over time?

The inductive analysis of professionals' interviews and survey exercises showed that qualities of SNS relate to children's abilities of HNC through two interrelated dynamics of change: *routinization* and *progression*.

*Routinization* fosters the development of children's HNC *within* abilities of HNC. It happens through reoccurring nature activities with similar qualities of SNS, but possibly across different socio-environmental situations. That is, the set of relations existing between different spatial natural features, social circumstances, and children's abilities generates qualities of SNS that develop certain abilities of HNC in depth. We selected few illustrative quotes from the interviews to exemplify the dynamic of routinization.

“*It is practice that gives them that skill. […] With the skiing for example, they can go downhill a hundred times and fall a hundred times. And the 101st time, they stand the whole hill down.”*

“*3 things that make nature play impactful: right kind of play, right kind of place, and the right kind of re-play […] is this frequency thing […] basically that it needs to happen, not necessarily everyday, but frequently.”*

*Progression* is the development of children's HNC *across* abilities of HNC. That is, the set of relations existing between different spatial natural features, social circumstances, and existing children's abilities generates qualities of SNS that allow children to develop abilities of HNC that they did not have before and broaden the development of their HNC; we selected few illustrative quotes from the interviews to exemplify the dynamic of progression.

“*Also, to build a positive feeling for nature you need to establish this sense of comfort already in young [children]- Being comfortable that you're able to, or dare to, carry your own backpack for example. That is sort of the first step. And then as you get a little bit older- To be able to walk a bit further away, maybe to another place.”*

“*First; you have to get accustomed to nature somehow, explore and discover, before you can play. […] Then you can start becoming interested in… Well, maybe practicing your agility by jumping from rocks or climbing tree logs or something.”*

“*[In a new place], you have to sort of start over, from this first phase [i.e., being comfortable].”*

**Figure 4:**
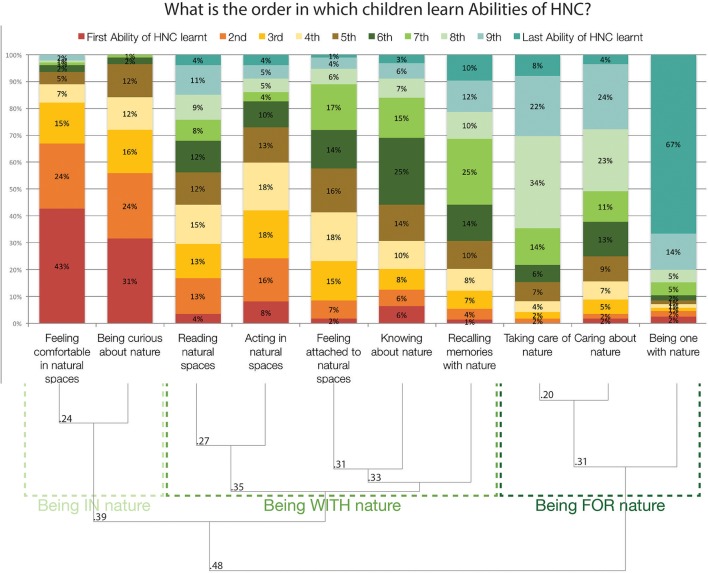
Results from the ordering exercise in the survey showing the progression of abilities of HNC in time. **Top:** each stacked column shows the percentages of professionals that placed as first, second, third, etc., or last each learnt ability of HNC. **Bottom:** the upside-down dendogram obtained from the hierarchical analysis of dissimilarity with height coefficients. The three obtained phases of HNC progression are also shown: being IN Nature, being WITH nature, and being FOR nature.

In order to fully appreciate the progression of children's abilities of HNC, we use a weighted average to rank the results from the ordering exercise in the survey. “Feeling comfortable in natural spaces” was found to be the first ability of HNC that children learn and “being one with nature” the last one (Figure 4). We also calculated a dissimilarity matrix on the results of the ordering exercise (min = 0.20, Mdn = 0.37, M = 0.41, max = 0.75), on which we performed hierarchical clustering to understand if the progression of abilities of HNC had clusters of development. Both the weighted averages and the dendrogram (agglomerative coefficient = 0.44) showed three clear clusters of abilities of HNC (Figure 4). The first cluster was defined by children's abilities of “feeling comfortable in natural spaces” and “being curious about nature,” so we termed it “*being IN nature.”* The second cluster is defined by children's abilities of “acting in natural spaces,” “feeling attached to natural spaces,” “reading natural spaces,” “knowing about nature,” and “recalling memories with nature,” so we termed it “*being WITH nature*.” The last cluster is defined by children's abilities of “caring about nature”, “taking care of nature,” and “being one with nature,” so we termed this cluster “*being FOR nature.”*

**Figure 5:**
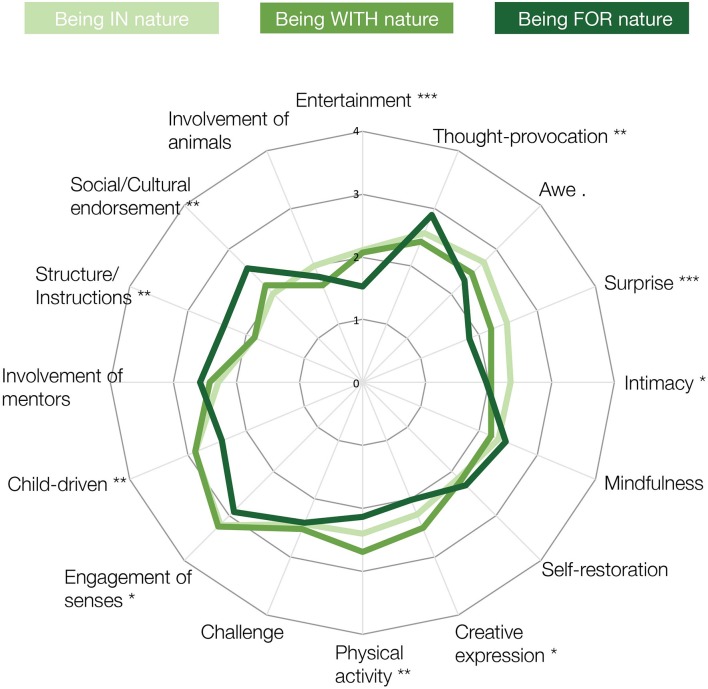
Configurations of qualities of SNS for the three phases of HNC progression (i.e. being IN nature, being WITH nature, being FOR nature). Qualities of SNS that are significantly different between phases of HNC progression are marked according to the following legend: ^***^p < 0.001; ^**^p < 0.01; ^*^p < 0.05; “.”: *p* < 0.1.

The dynamics between the qualities of SNS and children's abilities of HNC have been further explored using the results of the assessment exercise performed in the survey. This showed that in order to teach children specific abilities of HNC, professionals used nature activities that had different configurations of qualities of SNS (Figure 5). For example, professionals that intended to teach children to be capable of “*being IN nature”* performed nature activities that were mostly “child-driven,” characterized by high “engagement of senses”, “awe,” “physical activity,” but with little “structure/instructions.” Conversely, professionals who aimed to teach children to be capable of “*being FOR nature”* performed nature activities that were more characterized by “thought-provocation,” “social/cultural endorsement” and “structure/instructions” and less by “physical activity” or “entertainment.”

One-way ANOVA tests were conducted to compare the statistical influence of qualities of SNS on the three phases of HNC progression: “*being IN nature*,” “*being WITH nature,”* and “*being FOR nature*.” The results showed that many qualities of SNS were statistically different for different phases of abilities of HNC; that is, “entertainment” [*F*_(2, 396)_ = 6.811, *p* = 0.001], “thought-provocation” [*F*_(2, 396)_ = 4.665, *p* = 0.009], “intimacy” [*F*_(2, 396)_ = 3.85, *p* = 0.02), “awe” [*F*_(2, 396)_ = 3.841, *p* = 0.02], “surprise” [*F*_(2, 396)_ = 8.384, *p* = 0.0002], “physical activity” [*F*_(2, 396)_ = 0.467, *p* = 0.004], “social/cultural endorsement” [*F*_(2, 396)_ = 6.218, *p* = 0.002], “structure/instructions” [*F*_(2, 396)_ = 6.628, *p* = 0.001], “engagement of senses” [*F*_(2, 396)_ = 3.261, *p* = 0.04] and “child-driven” [*F*_(2, 396)_ = 5.916, *p* = 0.003]. For these qualities of SNS, Tukey *post-hoc* tests showed that there were significant differences between teaching children to be *in* or *with* nature and being *for* nature (Table 3). For instance, nature activities used in teaching children to be *for* nature were significantly less characterized by the qualities of SNS of “entertainment,” “physical activity,” and “child-driven,” but more defined by “thought-provocation,” “structure/instruction,” and “social/cultural endorsement.” Calculating Cohen's d on such differences also showed that the effect sizes were above average for educational research (*d* > 0.4) (Hattie, 2008) meaning that certain qualities of SNS were considerably more, and some less, important to nurture specific phases of HNC progression.

**Table 3:** Results of Tukey *post-hoc* test and Cohen's d for the qualities of SNS that were significantly different between the three phases of HNC progression (i.e., being IN nature, being WITH nature, being FOR nature).

**Qualities of SNS**	**Being IN nature**	**Being WITH nature**	**Being IN nature**	**Being FOR nature**	**Being WITH nature**	**Being FOR nature**
	***M*_in_ ± *SD*_in_** ***N*_in_ = **144****	***M*_with_ ± *SD*_with_** ***N*_with_ = **162****	***M*_in_ ± *SD*_in_** ***N*_in_ = **144****	***M*_for_ ± *SD*_for_** ***N*_for_ = **93****	***M*_with_ ± *SD*_with_** ***N*_with_ = **162****	***M*_for_ ± *SD*_for_** ***N*_for_ = **93****
Entertainment	2.1 ± 1.37	2.06 ± 1.26	2.1 ± 1.37	1.52 ± 1.26	2.06 ± 1.26	1.52 ± 1.26
			CI_0_._95_ = −0.7, −0.17^**^d = −0.44		CI_0_._95_ = −0.69, −0.17^**^d = −0.043	
Thought-provocation	2.58 ± 1.2	2.42 ± 1.16	2.58 ± 1.2	2.88 ± 1.02	2.42 ± 1.16	2.88 ± 1.02
					CI_0_._95_ = 0.16, 0.67^**^d = 0.41	
Awe	2.72 ± 1.2	2.46 ± 1.16	2.72 ± 1.2	2.29 ± 1.3	2.46 ± 1.16	2.29 ± 1.3
			CI_0_._95_ = −0.61, −0.08^*^d = −0.35			
Intimacy	2.36 ± 1.16	2.05 ± 1.36	2.36 ± 1.16	1.96 ± 1.3	2.05 ± 1.36	1.96 ± 1.3
			CI_0_._95_ = −0.59, −0.07^*^d = −0.33			
Surprise	2.48 ± 1.18	2.22 ± 1.17	2.48 ± 1.18	1.83 ± 1.35	2.22 ± 1.17	1.83 ± 1.35
			CI_0_._95_ = −0.79, −0.26^***^d = −0.52		CI_0_._95_ = −0.57, −0.06^*^d = −0.32	
Physical activity	2.4 ± 1.3	2.69 ± 1.33	2.4 ± 1.3	2.13 ± 1.27	2.69 ± 1.33	2.13 ± 1.27
					CI_0_._95_ = −0.69, −0.17^**^d = −0.43	
Engagement of senses	3.2 ± 0.97	3.23 ± 1.09	3.2 ± 0.97	2.9 ± 1.05	3.23 ± 1.09	2.9 ± 1.05
					CI_0_._95_ = −0.56, −0.05^*^d = −0.31	
Social/Cultural endorsement	2.01 ± 1.32	2.19 ± 1.24	2.01 ± 1.32	2.58 ± 1.15	2.19 ± 1.24	2.58 ± 1.15
			CI_0_._95_ = 0.19, 0.72^***^d = −0.45		CI_0_._95_ = 0.07, 0.58^*^d = −0.32	
Structure/Instructions	1.86 ± 1.23	1.86 ± 1.21	1.86 ± 1.23	2.38 ± 1.09	1.86 ± 1.21	2.38 ± 1.09
			CI_0_._95_ = 0.18, 0.71^***^d = 0.44		CI_0_._95_ = 0.19, 0.70^*^d = 0.45	
Child-driven	2.88 ± 1.1	2.87 ± 1.12	2.88 ± 1.1	2.42 ± 1.16	2.87 ± 1.12	2.42 ± 1.16
			CI_0_._95_ = −0.67, −0.15^**^d = −0.41		CI_0_._95_ = −0.65, −0.14^**^d = −0.40	

Cells with significant results (p < 0.05) with effect size d > 0.40 are highlighted. Legend for significance levels:

* p ≤ 0.05;

** p ≤ 0.01;

*** *p ≤ 0.001*.

## Discussing ACHUNAS: a framework to assess where and how children connect with nature

**Figure 6:**
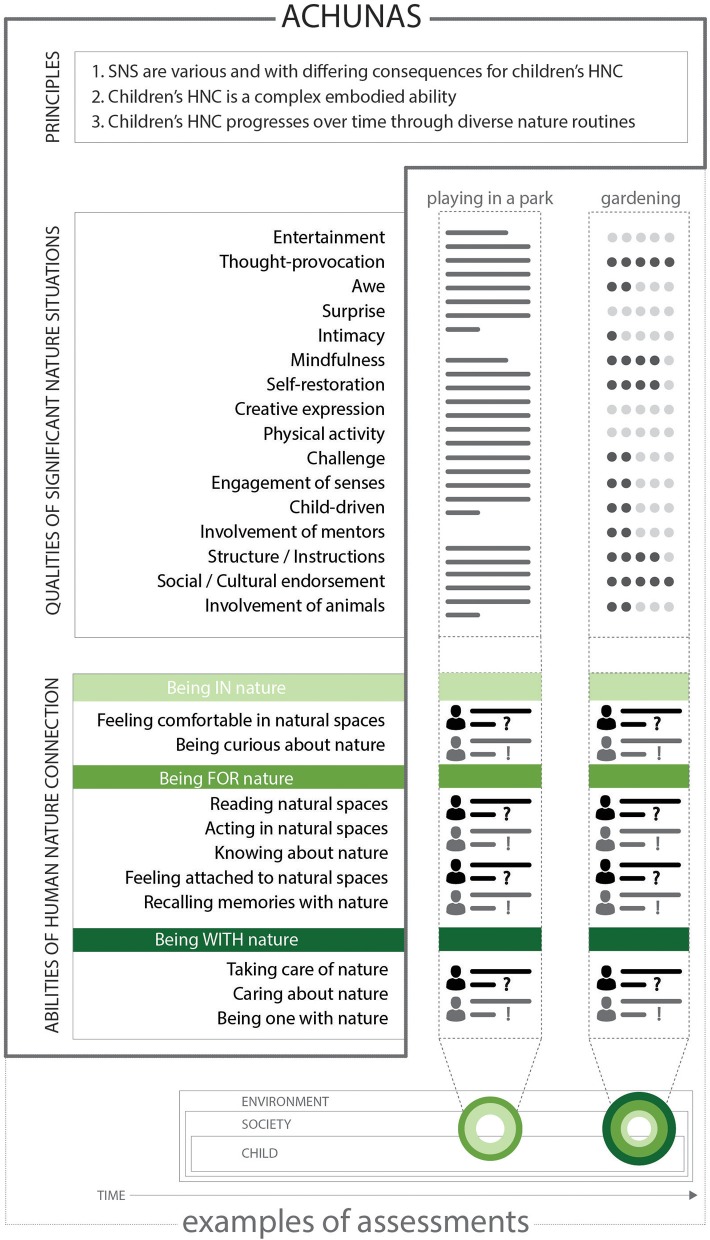
Assessment framework for Children's Human Nature Situations (ACHUNAS). ACHUNAS comprises the list of qualities of SNS, the list of children's abilities of human-nature connection, and three guiding principles. The figure shows the hypothetical assessment of two SNS (playing in the park and gardening) with different configurations of qualities of SNS and abilities of HNC.

The results shown in this paper form the Assessment framework for Children's Human Nature Situations (ACHUNAS) (Figure 6). ACHUNAS is composed of the list of qualities of SNS (Table 1); the list of children's abilities of HNC (Table 2); and three guiding principles (see section Guiding Principles of ACHUNAS). The lists of qualities of SNS and abilities of HNC outline *what* to assess to quantify or qualify the child-nature-connecting property of an environment, without defining *how* to perform the assessment itself. In line with transactional research, ACHUNAS avoids the rigid standardization of measurements across settings, and it solely highlights patterns of regularities across SNS and children's HNC (Altman and Rogoff, 1987). ACHUNAS is intended to be a flexible framework that allows practitioners and researchers to choose the assessment strategies, goals, and methods appropriate to their socio-environmental context. To allow the greatest flexibility while maintain integrity, three guiding principles are included as part of the framework. These principles give the boundaries of what SNS are, what children's HNC is, and how they relate to each other over time and are included as part of the framework. Together, the lists of qualities of SNS and abilities of HNC, and the three guiding principles form a comprehensive, transferable, and practical framework to guide the assessment of child-nature-connecting environments. Thus, ACHUNAS does not prescribe a set of methods to apply for a specific assessment, but it is a framework that outlines the criteria to apply when assessing child-nature-connectedness. ACHUNAS sets the framework to guide the assessment of where and how children connect to nature, but it does not impose a standard set of tools to measure child-nature-connectedness across all socio-environmental contexts.

### Guiding principles of ACHUNAS

This paper identified *three* crucial questions to address the problem of assessing child-nature-connectedness of environments: “what are the qualities of significant nature situations?” (RQ1), “what constitutes children's human-nature connection?” (RQ2), and “how do qualities of significant nature situations and children's human-nature connection relate to each other over time?” (RQ3). Below, we discuss the results that answer each of these questions with a principle. The *three* resulting principles set the boundaries of the assessment framework and guide how it should be interpreted and operationalized in the future.

#### Principle 1: significant nature situations are various and with differing consequences for children's HNC

Professionals identified sixteen qualities that make a nature situation significant for children's HNC (Table 1). Nature situations can be significant for children's HNC simply because they are entertaining, because they involve a personal engagement with animals, or because children feel free to engage in physical or artistic activities that activate their senses. However, it is most likely that several qualities of SNS co-occur in one significant nature situation. The configurations of qualities of SNS produced by the hierarchical clustering have shown the potential co-existence of six different kinds of SNS similar to some that have already been recognized in academia (Figure 2). Thought-provoking, awesome, and surprising SNS constituting “*environmental epiphanies”* are identified in the literature as “‘aha’ moments […] that shift the fundamental self-nature relationship” (Vining and Merrick, 2012, 1). Intimate, mindful, and self-restoring SNS constituting “*restorative experiences”* have a long academic history (Hartig et al., 1991; Kaplan, 1995) and are for example considered fundamental for healthy urban living (Hartig and Kahn, 2016). SNS characterized by “involvement of mentors,” “structure/information,” and “social/cultural endorsement” are equally recognized in environmental education (Nazir and Pedretti, 2016). Consequently, assessing the child-nature-connectedness property of environments implies assessing a variety of different SNS.

It is important to note that the diversity of SNS comes with a diversity of implications for children's HNC. Different configurations of qualities of SNS require and nurture children's HNC differently. Results from the survey have shown that the nature activities that professionals performed to teach children to care, take care, and be one with nature (i.e., to be FOR nature) are characterized by configurations of qualities of SNS that are statistically different from the other phases of HNC progression (see Figure 5, Table 3). As different qualities of SNS are used to promote different abilities of HNC it follows that some configurations of qualities of SNS are more important than others in developing certain abilities of HNC. During the assessment of where and how children connect to nature it is, therefore, important to remember that SNS are various and with differing consequences for HNC.

#### Principle 2: children's human-nature connection is a complex embodied ability

The professionals interviewed described HNC as a constellation of abilities of the mind and body that children learn. For example, professionals stated that children show HNC when they are “curious about nature”; which is visible in children when they show desire to know about nature as well as when they show the desire to physically explore natural spaces. Most abilities of HNC listed here simultaneously embody both actions and emotions, and are also embodied in particular socio-environmental contexts. During the interviews and in response to the survey, professionals widely remarked that the abilities that shape children's HNC are not only in relation to “nature” as an abstract concept, but they are also importantly related to natural physical spaces. Children's HNC exists when children are capable of “*feeling comfortable in natural spaces,” “acting in natural spaces,” “reading natural spaces,”* and ultimately “*feeling attached to natural spaces*.” These abilities of HNC show that children's HNC is rooted to tangible natural environments, and potentially specific ecological attributes, going beyond an abstract conceptualization of “nature” often used to assess people's relation with nature (Ives et al., 2017). Additionally, children's HNC shows attributes of complexity. The existence of different phases of HNC progression (Figure 4) and their differing relations to qualities of SNS (Figure 5) suggest that children's HNC as a whole cannot be solely understood as the sum of their individual abilities of HNC. Different configurations of abilities of HNC seem to play an important role in characterizing children's HNC as a whole. The second guiding principle to support the use of ACHUNAS is, therefore, that children's HNC is a complex embodied ability.

This principle answers the call of many in environmental psychology to integrate extra-psychological factors in models that aim to determine pro-environmental behaviors (Gifford, 2014; Gifford and Nilsson, 2014; Steg et al., 2014). Unlike previous academic work that presents mono-dimensional or disembodied understandings of the relationship that exists between people and nature, this paper demonstrates that professionals conceive children's HNC as a complex set of abilities embodied within mind, body, culture, and the environment. Despite socio-environmental contexts being considered obvious antecedents to encourage or constrain pro-environmental behaviors (Steg et al., 2014) academics have not yet systematically scrutinized their impact in encouraging pro-environmental behaviors (Steg and Vlek, 2009). The relational approach used here to develop ACHUNAS offers an alternative starting point that embraces and values professionals' embodied perspectives of children's HNC.

#### Principle 3: children's human-nature connection progresses over time through diverse nature routines

The thematic analysis of professionals' interviews showed that children's HNC progresses dynamically over time through routinization and progression like most other human abilities. In the survey, professionals suggested a model of development for children's HNC that begins with “feeling comfortable in natural spaces” and “being curious about nature,” and ends with “caring about nature” and “being one with nature” (Figure 4). The related hierarchical clustering showed three consecutive phases of HNC progression: being *in* nature, being *with* nature, and being *for* nature. That means that before being able to feel and act *for* nature (3rd phase) a child has to develop over time the ability to be *in* (1st phase) and *with* nature (2nd phase). In other words, before feeling care or concern for the environment, and before feeling responsible and motivated to act for it, a child has to at least feel at ease and comfortable in the natural elements of the outdoors. Children's ability to just enjoy and being curious about natural spaces is therefore a gateway to more profound forms of human nature relationships, but the progression across such abilities of HNC cannot be considered linear. Since these abilities are embodied in specific socio-environmental contexts, their progression is also context-dependent. As one of the interviewees stated “[In a new place], you have to sort of start over, from this first phase.” The three phases of HNC progression can indeed be seen as an indication of multiple states of equilibrium. This also implies that the development of children's HNC is likely to be characterized by potential threshold effects between one phase of HNC progression and another. For instance, only when a child is comfortable in nature in several social-environmental contexts can s/he then begin to know how to read specific environmental features. Abilities of HNC can indeed progress in depth (e.g., a child can be more or less curious about nature) and breadth (e.g., child can be curious about a garden, but not about a forest), and can nurture one another (e.g., a child who can be curious about a natural space might begin to feel attached to it). The third guiding principle to support the use of ACHUNAS is, therefore, that children's HNC does not grow linearly, but it progresses dynamically over time through the routinization of diverse qualities of SNS.

Current literature proposes models of development for HNC that grow linearly in independent and pre-identified psychological traits (Tam, 2013). The amount of time spent in a space is mentioned throughout the literature on sense of place as the most consistent predictor of attachment to it (Lewicka, 2011) and, similarly, the amount of time spent in nature is one of the most significant predictor of emotional affinity toward nature (Kals et al., 1999) and a crucial condition of any changes in children's HNC (Schultz and Tabanico, 2007). However, we are not familiar with any models that have explored how HNC develops and what is required for its progression over time. The development of children's HNC requires the reoccurrence of different qualities of SNS to progress. That is, children's HNC requires diverse nature routines provided by a wide variety of environmental features and by the involvement of diverse actors, collaborators, and institutions. Establishing significant and diverse nature routines responds to concerns in modern environmental education (Chawla and Cushing, 2007; Nazir and Pedretti, 2016), environmental conservation (Miller, 2005; Soga et al., 2015), and sustainable socio-ecological urban design (Colding and Barthel, 2013; Giusti et al., 2014; Marcus et al., 2016; Beery et al., 2017). As one interviewee highlighted: “3 things that make nature play impactful: right kind of play, right kind of place, and the right kind of re-play […] is this frequency thing.”

### Use and usefulness of ACHUNAS

The lists of abilities of HNC and qualities of SNS outline what to *quantify* or *qualify* when assessing child-nature-connecting environments. Similar to the lists of qualities used to assess the “child-friendliness” property of environments (Kyttä, 2006; Broberg et al., 2013), or the ANGELO framework used to assess the “obesogenicity” property of environments (Swinburn et al., 1999; Kirk et al., 2010), ACHUNAS is a framework that outlines a list-based set of criteria with the purpose of assessing the child-nature-connectedness property of environments. The lists of qualities of SNS and abilities of HNC give practitioners and researchers a frame of reference to identify where there are SNS and how they affect children's HNC. Eventually, the criteria proposed in ACHUNAS can be quantified using, for example, Likert scale surveys or other psychometric measurements. This would allow users to *quantify* the degree of “significance” of nature situations. Simultaneously, ACHUNAS is useful to understand what *kind* of SNS exist in the everyday landscapes and routines of children. The diversity of qualities of SNS and abilities of HNC listed provides the possibility to *qualify* the landscape in terms of the kind of SNS provided. For example, the green infrastructure might allow entertaining situations, but not nature situations that are child-driven or involving animals.

The above properties make ACHUNAS useful to assess, first, where significant nature situations are, and, second, how children connect to nature. Below, we give two hypothetical examples of assessments that operationalize ACHUNAS to exemplify its use in assessing child-nature-connecting environments.

#### Hypothetical examples of assessments using ACHUNAS

ACHUNAS can be used, for example, to assess the extent and type of “extinction of experiences” that many authors also believe undermines children's well-being (Soga and Gaston, 2016; Soga et al., 2016) and interest in nature conservation (Miller, 2005; Samways, 2007; Finch, 2008). Such assessments could be performed by using participatory observations, interviews, or PPGIS methodologies to examine which qualities of SNS exist, and which do not, in the everyday landscape of children. As an example, take an urban park in which children are freely allowed to play. Each item in the lists of qualities of SNS can be independently assessed according to the nature situations that are available in the park (see the assessment example “playing in the park” in Figure 6). In this hypothetical example, the evaluator has spent time in the park performing participatory observations of children describing nature activities with high levels of “entertainment,” but low levels of “thought-provocation.” Overall, the observations show that the nature situations available in this park are mostly characterized by the qualities of “child-driven” and “physical” activities with high “engagement of senses.” The evaluator then decides to interview children to understand their ability of “feeling comfortable in natural spaces,” “being curious about nature,” and so on. Once completed, the assessment provides the evaluator with useful information about what *kind* of nature experiences exist in the park, and, importantly, which ones are missing. In this example, the evaluator might conclude that organized activities and the introduction of animals might further nurture children's ability of “knowing about nature,” or “feeling attached to natural spaces.” In the above example, ACHUNAS specifies the list of criteria that the evaluator should follow to assess the environment, but the methods to do this (e.g., participatory observations and interviews) are chosen by the evaluator. For a more complete assessment, the evaluator might also consider principle 3 of ACHUNAS (i.e., children's HNC progresses over time through diverse nature routines) and assess how frequently children visit the park and how long they have exposure to nature activities for, or perform the same assessment for all the parks of a neighborhood or a city.

A second example of how ACHUNAS can be used is in the assessment of educational nature activities. This type of implementation would be particularly useful to educational programs connecting children to nature (see the assessment example “gardening” in Figure 6). Qualitative and/or quantitative methods such as interviews, observations, and questionnaires could be applied to study the presence and extent of the different qualities of SNS and abilities of HNC. In this hypothetical example, the project aims to teach children how to grow their own food, and researchers use a questionnaire to ask practitioners to rank how important each of the qualities of SNS are when children garden (this example has strong similarities with the assessment exercise performed by professionals in the survey). The final assessment shows that while gardening “thought-provocation” and “social/cultural endorsement” are high, but that “entertainment” is low. By interviewing the children, researchers also find that despite children being able to “take care of nature” their level of ability to “be curious about nature” while gardening is low. Researchers would conclude that gardening, as it is performed by this educational program, is likely to be insufficiently entertaining to nurture children's curiosity about nature. Researchers might then recommend that practitioners introduce an element of play during gardening activities, or to foster children's sense of belonging to the garden by making them choose a name for different sections of the garden. As in the previous example, ACHUNAS provides the lists of qualities of SNS and abilities of HNC which should be assessed, and the researchers select the method in which to do so (e.g., questionnaires and interviews). Using ACHUNAS in this way allows practitioners to develop more holistic and comprehensive nature activities, and it helps to bridge the gap between theory and practice that constitute a major obstacle in current environmental education (Chawla and Cushing, 2007; Finch, 2008; Nazir and Pedretti, 2016).

#### Comprehensiveness, transferability, and practicality

Of the 275 professionals responding to the survey (from more than 200 organizations in 22 countries), 74% considered the list of sixteen qualities of SNS to be “very” or “fully comprehensive” to assess *all* the qualities of nature activities that connect children to nature. During the survey, every single quality of SNS listed was considered “very important” or “essential” to assess a considerable percentage of nature activities that have the potential to connect children to nature; from 35% for “involvement of animals” to 78% for “engagement of senses” (Figure 1). The same can be said for the list of abilities that constitute children's HNC. The list of abilities of HNC was found to be at least “somewhat comprehensive” by 99.3% of the professionals responding our survey with the majority of professionals (63%) stating that it was “very comprehensive.” Additionally, a large majority of respondents agreed that every single ability of HNC listed indicated some form of connection to nature in children; from 79% agreeing with “being one with nature” to 92% agreeing with “being curious about nature” (Figure 3). These results, the mixed-method methodology, and the heterogeneous group of professionals ensure that the lists of qualities of SNS and abilities of HNC generated in this paper are sufficiently comprehensive of all relations that indicate children's HNC, transferable across different children and cultures, and can be practically implemented by practitioners and researchers in order to identify where children's SNS are.

#### Limitations of ACHUNAS

We identify three limitations of ACHUNAS to assess child-nature-connecting environments. The first limitation is the completeness and generalizability of ACHUNAS. 63% of professionals found the abilities of HNC “very comprehensive” and 67% of them thought the same for qualities of SNS, but only 7% of them found these lists to be “fully comprehensive.” This shows that ACHUNAS might not yet integrate all the qualities that can categorize an environment as more or less child-nature-connecting and, therefore, the lists of qualities of SNS and abilities of HNC require formal validation. Further research is required to test the lists for convergence or divergence. For generalizability, cross-cultural assessments are needed to understand if certain qualities of SNS and abilities of HNC are more suitable than others to assess specific socio-cultural contexts. Despite having tested ACHUNAS on a very international and heterogeneous group of respondents, the framework lacks the contributions of non-English speaking professionals and the potentially valuable input from indigenous communities.

Second, because of the nature of ACHUNAS, the framework does not suggest which method is the most suitable to assess each item in the lists. ACHUNAS highlights *what* to assess, but it does not provide guidance on *how* to assess child-nature-connectedness. Comparability across assessments that operationalize ACHUNAS with different methodologies might, therefore, be limited. Defining standard operationalizations of ACHUNAS through trandisciplinary collaborations would greatly increase the comparability of results across different socio-environmental contexts.

Third, ACHUNAS is a framework built upon professionals' understanding of children's HNC. The results are therefore a representation of a multitude of adults' perspectives and lack the direct input of children's insights. In order to further improve the validity of ACHUNAS as a framework, interviews of children and self-reporting methodologies performed with children can be implemented to better integrate children's perspectives into the ACHUNAS framework. In pursuing this endeavor, we also see the potential contribution of children with differing socio-demographic backgrounds.

### Future research directions for child-nature-connecting environments

We identified three future research directions that have great potential to promote the design of nature-connecting human habitats or to inform educational curricula that promote children's HNC. The most obvious is operationalizing ACHUNAS to assess where and how children connect to nature. Implementing standard operationalizations of ACHUNAS would allow understanding which relations of children's abilities of HNC and socio-environmental features afford qualities of SNS. This would be a further step forward in unveiling the ecological properties that can categorize an environment as more or less child-nature-connecting. Furthermore, such knowledge would be a stepping stone toward the design of nature-connecting human habitats.

A second area of research of fundamental importance is the analysis of interdependencies between qualities of SNS and abilities of HNC. The fundamental unit of analysis in ACHUNAS is at the confluence of children's abilities of HNC, qualities of SNS, and the socio-environmental context. The results shown above already indicated that certain configurations of qualities of SNS significantly relate to certain abilities of HNC (see Table 3). However, the current understanding of these linkages are preliminary and they lack temporal dimensions such as frequency or duration. A better understanding of the effects that routinized sets of qualities of SNS have on children's abilities of HNC would better inform the design of nature-connecting human habitats as well as pedagogical curricula that aim to connect children to nature.

Lastly, it is plausible that children's HNC may vary not just in relation to SNS and nature routines, but also with respect to the child's cognitive, emotional, moral, and physical stages of development. Although this has not been the goal of this paper, we recognize that studying the progression of HNC in relation to children's stages of development is a promising area of research that could directly promote comprehensive and holistic curricula to nurture children's HNC. For the sake of facilitating such academic endeavor, we believe that Piaget's stages of development (Piaget, 1960), Vygotsky's theory of Zones of Proximal Development (Chaiklin, 2003), Bronfenbrenner's theory of child development (Bronfenbrenner, 1979), situated and social learning (Lave and Wenger, 1991), and embodied cognition (Chemero, 2009) might be useful theories to investigate some of these unknowns.

### Implications for sustainable urban design and environmental education

The results of this study have obvious implications for the practice of sustainable urban design and environmental education. It is common for urban green infrastructure to be promoted and developed for biophysical management, e.g., stormwater management, flood control, urban cooling, reduction in carbon emissions etc., but its role in the development of children's HNC has been so far largely ignored. For example, access rights, spatial accessibility, ecological and biological diversity are just some of the variables of the green infrastructure that can promote children's nature routines and their HNC; all of which are already intentionally designed in the human habitat. Whether intentional or incidental, recurring experiences of nature situated in the everyday habitat of children are opportunities to develop their abilities of HNC (Giusti et al., 2014; Marcus et al., 2016; Beery et al., 2017; Samuelsson et al., 2017). Nature-based solutions provide direct benefits for public health, improve living conditions, and build resilience to climate and environmental change (Colding and Barthel, 2013; Hartig and Kahn, 2016). The results of this study show that they can also be considered in sustainable urban design for the transgenerational establishment of sustainable futures.

As environmental educators have already acknowledged, promoting children's HNC also means facilitating frequent nature activities and the results of this study confirm this position (Chawla and Cushing, 2007; Finch, 2008; Nazir and Pedretti, 2016). ACHUNAS offers the possibility to assess the significance of such frequent nature activities, to compare them, to identify which phase of HNC progression children are in, or to better tailor nature activities to suit the intended educational ability of HNC. In so doing, ACHUNAS is a first step toward the assessment of best practices to nurture children's HNC across organizations, cultures, and geographical locations. More broadly, assessing nature activities using ACHUNAS provides a preliminary form of curricula evaluation when the pedagogical goal is to connect children to nature. Conclusive monitoring methods developed from the ACHUNAS framework would allow reliable comparison within and across programs and the identification of discrepancies between intended and effective outcomes. This is a stepping stone to understand if such programs have potential to nurture future sustainable personal and collective behaviors. In summary, we believe ACHUNAS is a step forward in creating a reliable pedagogical curriculum capable of connecting children to nature.

ACHUNAS is a starting point for cross-fertilization between different disciplines interested in child-nature-connectedness. The academic literature on HNC has been systematically divided into epistemological silos (Ives et al., 2017), but the embodied approach to HNC used for ACHUNAS provides fertile ground to integrate, extend, and apply these different branches of empirical evidence. Several abilities of HNC in ACHUNAS such as “feeling comfortable in,” “feeling attached to,” “recalling memories in,” and “caring about” natural spaces are also founding elements of sense of place literature in human geography (Hernández et al., 2007; Lewicka, 2011). On the other hand, “knowing about nature” and to an extent “being one with it” are recognized components of modern environmental education (Nazir and Pedretti, 2016). The embodied nature of ACHUNAS allows these established disciplinary grounds to be brought together and set side by side with professionals' understanding of children's HNC. The use of ACHUNAS will allow these disciplines to gain further practical application and provide practitioners with the possibility to draw on the solidity of peer-reviewed literature.

## Conclusion

In this study, through the use of inductive thematic analysis and a practitioner survey we identified and tested three components of an Assessment framework for Children's Human Nature Situations that we called ACHUNAS (Figure 6). First, we identified 16 qualities of SNS that characterize a nature situation with the potential to “connect” children to nature. Second, we documented a list of 10 abilities of human-nature connection that expresses the nuances of children's human-nature connection. Third, we defined three principles that frame the applicability of these lists: (1) significant nature situations are various and with differing consequences for children's human-nature connection; (2) children's human-nature connection is a complex embodied ability; and (3) children's human-nature connection progresses over time through diverse nature routines. Together, these findings form a comprehensive framework that outline what to quantify or qualify when assessing “child-nature connecting” environments.

Questions like “where children connect with nature?” and “what kind of nature experiences are missing from cities?” are central to the design of nature-connecting human habitats as much as to the generation of sustainable futures. ACHUNAS is sufficiently comprehensive, transferable, and practical to provide a starting point to answer these questions and to guide the assessment of where and how children connect to nature. It can be operationalized to assess the extent and typology of “extinction of experience” and inspire the design of nature-based human habitats that also connect children and people to nature. Similarly, it can be used to evaluate curricula that aim to connect children to nature, providing useful information to such educational programs. Overall, ACHUNAS is a transdisciplinary framework that allows cross-fertilization and integration of knowledge across different academic disciplines and makes it useful to practitioners interested in promoting child-nature-connecting environments and children's HNC. In conclusion, ACHUNAS provides a starting point to classify a social-ecological system as more or less child-nature-connecting.

## Ethics statement

This study was carried out in accordance with the recommendations “ESPA Ethics Principles and Procedure” produced by the Directorate of the Ecosystem Services for Poverty Alleviation, with written informed consent from all subjects. All subjects gave written informed consent in accordance with the Declaration of Helsinki. The protocol was approved by the ethical committee at the Stockholm Resilience Centre.

## Author contributions

MG: Work conception, research design, data collection, data analysis, data interpretation, paper arrangement and revision, writing, and submission; US: Data collection, data analysis, data interpretation, paper arrangement and revision, and writing; CR: Data interpretation, research design, paper arrangement and revision, and writing; TB: Data interpretation, paper arrangement and revision, and writing.

### Conflict of interest statement

The authors declare that the research was conducted in the absence of any commercial or financial relationships that could be construed as a potential conflict of interest.
